# Molecular Network Basis of Invasive Pituitary Adenoma: A Review

**DOI:** 10.3389/fendo.2019.00007

**Published:** 2019-01-24

**Authors:** Qi Yang, Xuejun Li

**Affiliations:** Department of Neurosurgery, Xiangya Hospital, Central South University, Changsha, China

**Keywords:** angiogenesis, endocrinology, invasiveness, molecular network, pituitary adenoma

## Abstract

Cases with pituitary adenoma comprise 10–25% of intracranial neoplasm, being the third most common intracranial tumor, most of the adenomas are considered to be benign. About 35% of pituitary adenomas are invasive. This review summarized the known molecular basis of the invasiveness of pituitary adenomas. The study pointed out that hypoxia-inducible factor-1α, pituitary tumor transforming gene, vascular endothelial growth factor, fibroblast growth factor-2, and matrix metalloproteinases (MMPs, mainly MMP-2, and MMP-9) are core molecules responsible for the invasiveness of pituitary adenomas. The reason is that these molecules have the ability to directly or indirectly induce cell proliferation, epithelial-to-mesenchymal transition, angiogenesis, degradation, and remodeling of extracellular matrix. HIF-1α induced by hypoxia or apoplexy inside the adenoma might be the initiating factor of invasive transformation, followed with angiogenesis for overexpressed VEGF, EMT for overexpressed PTTG, degradation of ECM for overexpressed MMPs, creating a suitable microenvironment within the tumor. Together, they form a complex interactive network. More investigations are required to further elucidate the mechanisms underlying the invasiveness of pituitary adenomas.

## Introduction

Cases with pituitary adenoma comprise 10–25% of intracranial neoplasm (1) and has a prevalence rate of about 17% in the general population (2). Most of the adenomas are considered to be benign. The symptoms of pituitary adenomas contain two major aspects, endocrine related and tumor occupying symptoms, the former differs according to the various hormones that get involved, the later one includes vision loss and headache. Some adenomas found accidentally on an MRI scan also show no clinical symptoms at all. The diagnosis of pituitary adenoma requires both imaging evidence and serum hormone level. About 35% of pituitary adenomas are invasive (3), which are defined and graded by the extent of tumor invading the adjacent sphenoid sinus and cavernous sinus. Invasive pituitary adenomas not only are more difficult to achieve total resection, but also have a higher recurrent rate after standard surgery compared to benign ones.

A few classification systems are available for evaluating invasive pituitary adenomas to aid surgical planning, including Hardy classification, Wilson–Hardy classification, and Knosp classification. Invasive pituitary adenomas are more difficult to surgically remove, and most of the time, they require surgical resection for the relatively more severe symptoms. An invasive pituitary adenoma was considered synonymous with an aggressive adenoma in a number of studies, moreover, aggressive ones are usually macroadenoma (4). However, some scholars (4, 5) preferred to regard an aggressive adenoma as a separated type that displays more aggressive clinical progression despite of the tumor size and should be diagnosed based on the elevated immunoreactivity of Ki-67 and P53 over more “benign” types of pituitary adenoma using tissue immunohistochemistry. Ki-67 is a biomarker widely used to evaluate cell proliferation. P53 is also a biomarker that indicates malignancy and invasiveness when found strongly positive on tumor tissue immunohistochemistry. The 4th edition of WHO classification of pituitary tumors has removed the term atypical pituitary adenoma (APA) for the difficulty and inconsistency in determining proper cutoff of the diagnostic criteria being used before (4, 6–9), in the previous 3rd version, APA is defined as tumors that display invasive growth, Ki-67 index >3%, extensive nuclear staining for p53 and elevated mitotic activity (10), which is vague. So the 4th edition of WHO classification of pituitary tumors has also suggested that the grading of aggressive pituitary adenoma should be evaluated on an individual case basis with criteria mentioned above (7). In some ways, aggressive, and invasive pituitary adenomas can be different in clinical behavior, but they largely share the same molecular basis in terms of malignancy and invasiveness. P53 protein and Ki-67 protein are common biomarkers shared between kinds of tumors, the difficulty (i.e., inconsistent criteria) of using them in grading of pituitary adenoma means that we need some more accurate biomarkers to better distinguish them from benign pituitary adenomas.

Compared with non-invasive pituitary adenomas, those with invasive behavior are difficult to tackle with. Therefore, it is necessary to identify their causes. This review summarized the known molecular basis of the invasiveness of invasive pituitary adenomas, providing insights for further exploration in this field.

## Vascular Endothelial Growth Factor and Related Factors in an Invasive Pituitary Adenoma

**Figure 1:**
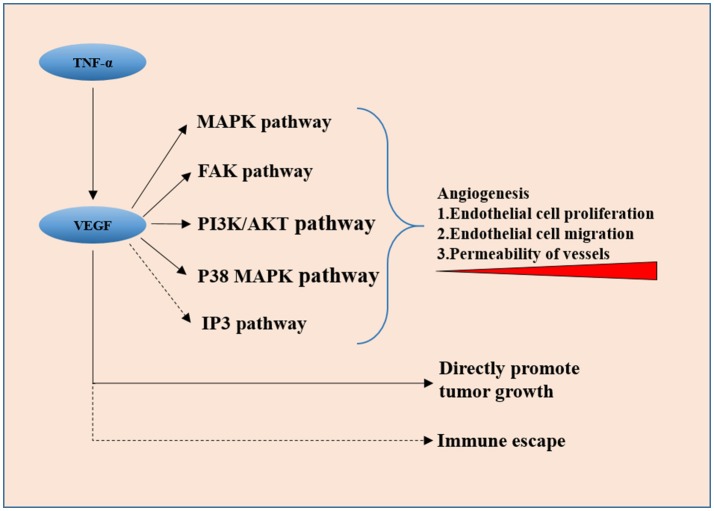
VEGF secreted by tumor cells promotes neovascularization via downstream pathways including the MAPK signaling pathway, FAK pathway, PI3K/Akt pathway, and p38 MAP kinase pathway, it also directly promotes tumor cell proliferation. IP3 pathway and VEGF-induced immune escape might be involved in invasiveness of the pituitary adenoma.

Increased angiogenesis is found to be essential for the invasiveness and spread of many types of tumors including invasive pituitary adenoma. Invasive macroprolactinomas and non-functional adenomas were more vascular compared with non-invasive ones (11) on surgically removed human pituitary adenomas. As shown in Figure 1, angiogenesis in the tumor is a complex and dynamic process involving the endothelial matrix degradation, proliferation, and migration of endothelial cells, and remodeling of the vascular basement membrane. In general, whether pituitary adenomas are more vascular is still controversial (12–14), but when it comes to invasive or aggressive pituitary adenomas and carcinomas, it is safe to say that angiogenesis is essential (15).

### Vascular Endothelial Growth Factor

Vascular endothelial growth factor (VEGF) is proved to be the key factor in angiogenesis in many human tumors. Overexpression of VEGF in clinical samples of invasive pituitary adenomas is observed by many scholars (16). Meaning that VEGF could also be used as an independent prognosis-predicting factor except for Ki-67 and P53. There is a significant relationship between the expression of VEGF and apoplexy of pituitary adenoma (17), which, from another point of view, is an indicator of rapid tumor vascular growth. VEGF secreted by tumor cells promotes neovascularization via downstream pathways including the MAPK signaling pathway (18), FAK pathway (19), PI3K/Akt pathway (20), and p38 MAP kinase pathway (21), directly stimulating tumor cell proliferation (22). The function and the role of IP3 signaling pathway, which is also a classic downstream pathway of VEGF, in the invasiveness of pituitary adenoma are not clear yet. This could be a new entry point for the investigation of VEGF and its peripheral signaling pathways. The activation of these pathways promotes angiogenesis in many ways, including vascular endothelial cell proliferation, migration, and increase in the permeability of newly formed vessels. The controversial results on the microvascular density of pituitary adenomas indicate that VEGF has a more direct and pivotal role in tumorigenesis and invasiveness rather than just in angiogenesis because VEGF-related pathways have been found to directly promote tumor cell proliferation in other cancers recently (23). The immune escape modulated by VEGF was reported (24) and left untested in pituitary adenomas.

### Tumor Necrosis Factor α

Some other molecules involving angiogenesis have shown a regulatory effect on the expression of VEGF. Tumor necrosis factor**-** α (TNF-α) has been reported to upregulate the expression of VEGF and matrix metalloproteinase-9 (MMP-9) in rodent cell line MMQ. In human pituitary adenoma surgical specimens, higher expression levels of TNF-α, VEGF, and MMP-9 were found in hemorrhagic adenomas than in non-hemorrhagic ones. Also, the expression levels of both VEGF and MMP-9 were positively correlated with TNF-α (25). Pituitary apoplexy can cause secondary hypoxia of the tumor tissue, so the elevation of TNF-α and VEGF expression might just be the self-saving struggle under extreme conditions.

### Hypoxia-Inducible Factor-1α

Hypoxia-inducible factor-1α (HIF-1α) is able to regulate VEGF in pituitary adenomas and other human tumors. More than a decade ago, the apoptotic protective function of HIF-1α in human pituitary adenoma cell line HP75 under hypoxic conditions via a knockdown experiment was observed (26). VEGF was then confirmed to be activated by HIF-1α (27), but the HIF-1α overexpression model of rodent MMQ cell culture showed a higher apoptotic rate compared with the control, which contradicted the results of knockdown experiments in the human cell line. RWD-containing sumoylation enhancer (RSUME), a stabilizer of HIF-1α under hypoxia (28), is reported to upregulate VEGF *in vitro*, substantiating the interaction between HIF-1α and VEGF (29). There is overexpression of RSUME, HIF-1α, and VEGF-A in invasive pituitary adenoma surgical specimens compared with non-invasive ones, confirming RSUME as an upstream regulator of expression after the HIF-1α knockdown. Furthermore, the RSUME knockdown rodent AtT-20 cell line demonstrated a more invasive behavior (30). All these experiments displayed the whole picture of an invasiveness-inducing pathway of RSUME–HIF-1α-VEGF.

Notably, the von Hippel-Lindau gene-related protein (pVHL) is a known negative regulator of HIF-1α. Its low expression with a high expression level of VEGF leads to a higher recurrence rate and more aggressive behavior (31). A study reported an aggressive GH-PRL pituitary adenoma in a young patient with a VHL gene missense mutation (32). All these further confirm the importance of VEGF-related pathways in the invasiveness and aggressiveness of pituitary adenomas.

### Fibroblast Growth Factor-2

It has been shown that fibroblast growth factor-2 (FGF-2), a growth factor, also promotes vascular endothelial cell proliferation and differentiation similar to VEGF. It is an upstream upregulator of VEGF in human vascular endothelial cells (33) and rodent GH3 pituitary cell line (34). However, FGF-2 had no significant modulatory effect on VEGF at the transcription level in human pituitary cell line HP75 (35). The overexpression of fibroblast growth factor receptor 1 (FGFR1), a receptor of FGF-2, is also closely related to the invasiveness of pituitary adenomas (36). Additionally, FGF-2 had significantly reduced expression levels in male and female prolactinoma patients (37), together with the study showing that FGF-2 had no regulatory effect on cell proliferation (38) and no significant modulating effect on VEGF at the transcription level in human gonadotrophic cell line HP75 (35), indicating FGF-2 might only be effective only in early stages of the development of pituitary adenoma, moreover, only in human prolactinoma.

### Exploiting VEGF-Related Pathways in Treating Pituitary Adenoma

Advances in molecular biology have provided a better understanding of invasive pituitary adenomas, improving clinical prognosis. Many successful attempts had been made to exploit VEGF-related pathways in treating pituitary adenomas, from directly targeting VEGF (39–42) to its upstream pathways (43, 44), and to other related molecules (45).

## Pituitary Tumor Transforming Gene

First cloned in 1997 (46), the pituitary tumor transforming gene (PTTG) is a known oncogene and upregulator of VEGF (47). The relationship between PTTG and VEGF was later elucidated with the findings that the PTTG upregulate and co-locate with VEGF, thus indirectly promoting angiogenesis in pituitary adenomas.

The PTTG1 is also called securin protein, which counters the function of separin. The degradation of PTTG1 triggers the anaphase of mitosis. Separin then promotes chromosome segregation (48). In human pituitary surgical specimens, invasive pituitary adenomas had the highest level of PTTG followed by non-invasive ones. And the PTTG doesn't express in a normal pituitary tissue (36, 49). Other researchers also reported PTTG as an indicator of both invasiveness and aggressiveness of pituitary adenomas in clinical studies (50–52). A meta-analysis on 15 cohorts of a total 752 patients with a pituitary adenoma further corroborated the relationship between PTTG and invasiveness in pituitary adenomas (53). The elevated PTTG expression level is expected to directly increase cell proliferation and chromosomal instability (54), implying enhanced tumor invasiveness.

Apart from the relationship with VEGF, the PTTG has a wide interaction spectrum with many genes and molecules related to survival, mitogenesis, tumor growth, and invasion. Estrogen receptor α (ERα), a nucleus-located receptor and the mediator of estrogen, is positively related to the invasiveness of human pituitary adenomas (55, 56) and has a significantly higher expression in male patients with prolactinoma than in normal pituitary tissue (37). In the present study, male patients had much higher serum PRL level and much larger tumor volume compared with female patients. The expression of ERα was also elevated in female patients, but not significantly. Estrogen is the first discovered inducer of PTTG, the transcription and translation levels of the PTTG both increased within 48 h after estrogen administration in a “synced” fashion to the estrogen administration in a study using prolactinoma rat model, and under the administration of estrogen, PTTG, FGF-2, VEGF showed the same expression pattern, showing that estrogen is an inducer of PTTG. In the same study, microscopic observation of the pituitary tumor showed progressive neovascularization and remodeling of the extracellular matrix (ECM) (57). All these findings elucidated that PTTG, FGF-2, and VEGF might act in synergy from the early development to increase the invasiveness and angiogenesis of pituitary adenomas, especially prolactinoma (58) and growth hormone–secreting adenomas (59). Connexins (Cx) is a protein family forming the gap connections of cells, expression changes of which between tumor and normal tissue have been reported in many types of cancers (60). Among them, Cx43 is ubiquitously expressed in vertebrates and is considered a tumor suppressor, in most cancer types like testis cancer (61, 62), breast cancer (63, 64), and colorectal cancer (65) tumor cells tend to have lower expression of it, but that is not the case in prolactinoma. Experiments on rat prolactinoma model has shown us that estrogen can induce increasing of gap junctions and of course Cx43, and the silencing of Cx43 could attenuate estrogen-induced up-regulation of PTTG (66). Cx43 might play an import part in tumorigenesis of prolactinoma, the relationship between Cx43 and VEGF, HIF-1α in pituitary adenomas requires future investigation. It is worth mentioning that the PTTG can upregulate the expression and secretion of MMP-2 in HEK293 cells (67), MMP-2 is capable of inducing invasiveness in pituitary adenomas. It could be the same for the PTTG in pituitary adenomas. The clarification of this would be worthwhile because both PTTG and MMPs are potentially valuable therapeutic targets.

## Degradation and Remodeling of ECM by Matrix Metalloproteinases Family

Matrix metalloproteinases (MMPs) are a group of calcium-dependent zinc-containing endopeptidases with the ability to degrade basement membrane and ECM. Together with the tissue inhibitor of metalloproteinases (TIMPs), they are the essential elements in the stability and remodeling of ECM (11). A dynamic balance is maintained between MMPs and TIMPs. The major types of MMPs involved in pituitary adenoma invasion can be classified into collagenases (MMP-1), gelatinases (MMP-2 and MMP-9), stromelysins (MMP-3), and membrane type (MMP-14) according to their function and location. TIMPs (TIMP-1, TIMP-2, TIMP-3, and TIMP-4) and reversion-inducing cysteine-rich protein with Kazal motifs (RECK) act as inhibitors, and extracellular matrix metalloproteinase inducer (EMMPRIN) acts as the inducer of MMPs.

MMP-9 is the first matrix metalloproteinase found to have a significantly higher expression level in pituitary adenomas invaded to cavernous sinus (68). However, TIMP-1 was undetectable by immunochemistry staining in all samples (69). The correlation between MMP-9 overexpression and invasiveness of pituitary adenomas has been verified by many researchers in human pituitary adenoma specimens (70–75) as well as cell lines (76). Later studies showed that high expression levels of EMMPRIN (77, 78), MMP-2 (71, 75, 79), and MMP-14 (80, 81) and low expression levels of TIMP-2 (82, 83), TIMP-3 (82, 84), and RECK (85) were also correlated with invasiveness. There is a report that found TIMP-2 have higher expression in more patients of invasive prolactinomas then non-invasive ones (74), most of the aforementioned studies were performed on patients with prolactinoma or mixed patients of all secreting types, the contradicting results of TIMP-2 indicating that different types of pituitary adenoma might have distinct signaling pathways regarding to invasiveness. However, no statistical difference in the MMP-9 expression level between invasive and non-invasive non-functioning pituitary adenomas could be found (86).

MMP-9 plays an important role in promoting invasiveness in many type of pituitary adenomas. A transcriptome analysis on somatotroph pituitary adenomas (87) identified genes having a differential expression pattern between the two groups depending on the invasiveness. Hepatocellular carcinoma, downregulated 1 (HEPN1) was found to be less expressed in invasive somatotroph pituitary adenoma. First found to be downregulated in hepatocellular carcinoma. HEPN1 can induce apoptosis when overexpressed in HepG2 (88). In rodent cell lines GH3 and GT1-1, it inhibited the expression of MMP-2 and MMP-9, resulting in reduced invasiveness (87). A transcriptome and proteome analyses on pituitary null cell adenomas, a subtype of non-functioning pituitary adenomas, by the same research team (89), identified that upregulated IL-6R/JAK2/STAT3 promoted invasiveness via MMP-9.

MMPs not only promote invasiveness by the degradation of ECM and the consequential release of various ECM-anchored growth factors (90, 91), other functions are also observed. Interfering with the expression of MMP-14 using shRNA could result in the reduced expression of PTTG, VEGF, and TGFβ in rodent AtT-20 cells (80), implying that MMPs would also directly promote tumor growth and angiogenesis. IL-17 and IL-17 receptors were positively related to MMP-19 in terms of expression levels. The levels were all elevated in invasive pituitary adenomas compared with non-invasive ones (92).

More recent studies have demonstrated the difference in genotyping of patients with pituitary adenoma; polymorphisms of MMP-9 (93) and promoter of MMP-1 (94) could affect invasive phenotype. Other proteases were also demonstrated to promote the invasiveness of human pituitary adenomas, including a disintegrin and metalloproteinase 12 (ADAM12) (81) and serine proteases urokinase-type plasminogen activator (uPA) (83). Interestingly, the same research team reported the involvement of ADAM12 in invasiveness. They later demonstrated that ADAM12 was also involved in epithelial-to-mesenchymal transition (EMT) (95).

Enhancer of zeste homolog 2 (EZH2) is widely involved in many cancers. It is a key catalytic component in polycomb repressive complex 2 (PRC2), which is responsible for the methylation modification of many development- and differentiation-related genes. It is therefore important in tumorigenesis of many human cancers (96). The overexpression of EZH2 in pituitary tumors was found to be related to invasiveness possibly via the upregulation of MMP-14 (97).

### Potentials of MMP Inhibitors in Treating Pituitary Adenoma

Efforts were made to evaluate the potential of MMP inhibitor in treating invasive and chemotherapy-refractory pituitary adenomas. Batimastat showed inhibitory effect on the rat prolactinoma model (98) by reducing the cell proliferation rate and promoting the apoptosis.

## EMT and Invasive Pituitary Adenoma

EMT is a process that increases the invasiveness of tumor characterized by the loss of epithelial-cadherin (E-cadherin) and the enhanced expression of transcription factor snail family transcriptional repressor 1 (SNAI1) gene (also referred to as Snail), transcription factor snail family transcriptional repressor 2 (SNAI2) gene (also referred to as Slug), forkhead box C1 (FOXC1), twist-related protein 1(TWIST1), neural cadherin (N-cadherin), and Vimentin.

In an early study employing immunohistochemistry on 30 pituitary adenomas, the semi-quantified immunoreactivity of E-cadherin level was not correlated with cavernous sinus invasion (99). A follow-up study with larger sample size and better methodology, using quantitative real-time polymerase chain reaction on cadherin 13 (CDH13) and immunohistochemistry on E-cadherin and β-catenin, demonstrated the expected significantly lower expression levels of E-cadherin (100–102), CHD13 (101), and β-catenin (100, 102) in invasive pituitary adenomas, resulting from a more frequently methylated CDH13 and E-cadherin genes (101). Also, a study reported the nuclear accumulation and translocation of E-cadherin (103), suggesting another possible mechanism for the less expressed E-cadherin in invasive pituitary adenomas.

In a microarray analysis of human somatotroph adenomas, epithelial splicing regulatory protein 1(ESRP1) was differentially expressed in two groups with relatively low or high transcription levels of E-cadherin; the results were validated with RT-PCR and *in vivo* experiment in GH3 cells. A gene set comprising ESRP1, PKP2, TP53, PERP, IRF6, ROBO1, BICC1, SPINT1, and of course, CDH1 (E-cadherin) was also found to have reduced expression levels (104). With the same sample set, they demonstrated that it was possible to accurately discriminate invasive pituitary adenomas from non-invasive ones using the binary tree analysis on a group of genes including ESRP1, CDH1, and CTNNb1. Therefore, the potential EMT and invasiveness promoting function of genes in this set makes them valuable targets worth further investigation. Among them, the expression level of ESRP1 was confirmed related to the invasiveness of prolactinoma and GH-secreting adenoma later (105).

A number of miRNAs were reported to regulate EMT. The overexpressed miR-133 could upregulate the expression of E-cadherin and downregulate the expression of N-cadherin and Snail (106). The overexpression of miR-132, miR-15a, and miR-16 could downregulate the expression of N-cadherin and TWIST1 genes (107). The expression of Slug was positively correlated with ERα and invasiveness in clinical pituitary adenoma specimens (56), showing that the ERα-Slug–E-cadherin pathway was vital in the invasiveness of pituitary adenomas. The overexpression of miR-133 suppressed invasion by downregulating the expression of transcription factor forkhead box C1 (FOXC1) in HP75 cells (106), implying the involvement of miR-133 in EMT; FOXC1 is a known promoter of EMT (108).

PTTG-induced EMT is an important mechanism of tumor invasiveness and metastasis in lung cancer (109) and ovarian cancer (110). However, its involvement in pituitary adenomas has not been elucidated yet.

## MiRNAs and Invasive Pituitary Adenoma

Available evidence shows that the levels of miR-24, miR-34a, miR-93 (111), miR-148-3p, miR-152 (112), miR-132, miR-15a, and miR-16 (107) are significantly lower in invasive pituitary adenomas (111) compared with non-invasive ones. The overexpression of miR-148-3p and miR-152 suppressed invasion by downregulating activated leukocyte cell adhesion molecule (ALCAM) in rodent GH3 cells (112). Also, the overexpression of miR-132, miR-15a, and miR-16 suppressed invasion by downregulating sex-determining region Y-box protein 5 (Sox5) gene in rodent GH3 cells (107). Some miRNAs are reported to have elevated expression levels in invasive pituitary adenomas. MiR-93-5p was overexpressed in invasive (113) corticotroph pituitary adenomas. The expression of miR-106b-5p, miR-93-5p, miR-93-3p, and miR-25-3p, as a cluster, is also positively correlated with invasiveness. The enhanced expression of miR-106b can induce invasiveness via PI3K/PTEN/Akt pathway and sequential overexpression of MMP-9 in HP75 cells (114). Using a miRNA microarray, many differentially expressed miRNAs in non-functioning pituitary adenomas was identified in a single study. The expression levels of miR-181b-5p, miR-181d, miR-191-3p, and miR-598 were upregulated, and the expression levels of miR-3676-5p and miR-383 were downregulated (115). Caveolin-1 (Cav-1) was reported to promote invasiveness via the EGR1/KLF5 pathway in GH3 cells. Its knockdown resulted in a cytoplasmic enrichment of EGR1, which then induced miR-145, miR-124, and miR-183 targeting FSCN1, PTTG1IP, and EZR, respectively (116). MiRNAs are critical in prompting invasiveness in pituitary adenoma, many molecules and pathways are involved, miRNA sequencing would be a proper method comprehensively identifying differentiatly expressed miRNAs, after which targets of these miRNAs can be predicted with bioinformatics tools, functions of them would then be validated with pertinence.

## Other Genes Involved in the Invasiveness of Pituitary Adenoma

In a prospective study on 94 patients with prolactinoma, A Disintegrin And Metalloproteinase With Thrombospondin Motifs 6 (ADAMTS6) and Collapsin Response Mediator Protein 1 (CRMP1) were found to be positively related to invasiveness, while the overexpression of PTTG, Cyclin B1(CCNB1), Aurora Kinase B(AURKB), and Centromere Protein E(CENPE) indicated both invasive and aggressive behavior (51), these genes are all mitosis or development related, they might be the key biological processes that transduce invasiveness transformation. A causative CDH23 gene mutation was identified in a family of familial pituitary adenoma and sporadic patients with this mutation have non-invasive phenotype (117). Secreted frizzled-related protein 1(sFRP1) and Wnt inhibitory factor 1(WIF-1) genes were found to be less expressed in invasive non-functioning pituitary adenomas (118). Also, transforming growth factor, beta receptor II(TGFβII), is less expressed in invasive non-functioning pituitary adenomas (119).

Epidermal growth factor-like domain multiple 7(EGFL7) has a higher level of cytoplasmic expression in invasive growth hormone–secreting pituitary adenomas (120), and is positively correlated with Notch2 and Dll3 in knockdown experiments on GH3 cells. Later the invasiveness reduction phenomenon was reported after the knockdown of EGFL7 in GH3 and GT1-1 cells *in vitro* (121), confirming EGFL7 as a valuable therapeutic target.

Recent reports implied that long non-coding RNAs (lncRNAs) were involved in invasiveness. The expression of lncRNA C5orf66-AS1 was downregulated and inversely related to invasiveness in pituitary null cell adenoma compared with normal pituitary and non-invasive ones (122). However, lncRNA H19 was upregulated in invasive growth hormone–secreting pituitary adenomas (123).

Epigenetic modification of certain genes has been proved to induce invasiveness in pituitary adenomas including P16, DAPK, and Rb1 (124–129), these genes could be new targets of therapy (130). High throughput sequencing of methylation status (i.e., ChIP-sequencing) in the future will hopefully provide us with the global view of the epigenome of pituitary adenomas.

Next-generation sequencing (NGS) is a powerful tool discovering new disease-related genes at a relatively low cost, especially RNA sequencing and whole-exome sequencing. Using NGS on invasive pituitary adenomas and 6 non-invasive pituitary adenomas, 15 genes with pathogenic mutations were identified (131), including EGFL7, LRP1B, MGAM, MAST4, DSPP, PRDM2, PRDM8, ZNF717, LRRC50, TRIOBP, MX2, DPCR1, PRB3, SPANXN2, and KIAA0226. They also reported that CAT, CLU, CHGA, EZR, KRT8, LIMA1, SH3GLB2, and SLC2A1 were invasion-related genes in non-functioning pituitary adenomas (132). And data mining on existing pituitary adenoma RNA sequencing data from the National Center for Biotechnology Information Gene Expression Omnibus identified invasion-associated genes (133), including CLDN7, CNTNAP2, ITGA6, JAM3, PTPRC, and CTNNA1. All these genes could be the critical cause for invasiveness, which needs further exploration.

## Conclusions and Perspective

Efforts were made to elucidate the molecular mechanisms of the invasiveness in pituitary adenomas, from the observation of possible biomarkers on tumor specimens to function verification experiments *in vitro*, and to recent application of multi-omics analysis. Yet, the whole picture is unclear because the molecular basis of invasiveness is highly complex involving multiple genes, proteins, and pathways. What makes it even more difficult is the fact that subtypes of pituitary adenomas are different in many ways rather than just the hormone they secrete.

**Figure 2:**
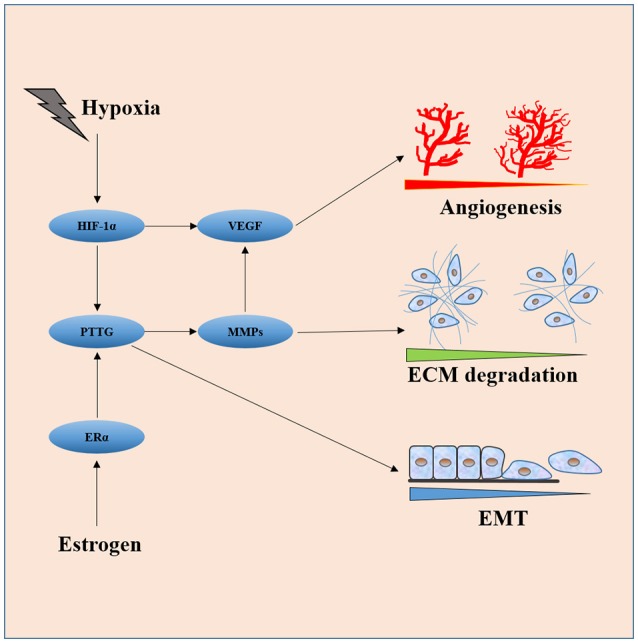
Interaction of core molecules with each other and their relationship with EMT, angiogenesis, and ECM degradation. HIF-1α induced by hypoxia or apoplexy inside the adenoma might be the initiating factor of invasive transformation, followed with angiogenesis for overexpressed VEGF, EMT for overexpressed PTTG, degradation of ECM for overexpressed MMPs.

Luckily, studies in the last two decades provide some clues in this regard. The key nodes of the invasiveness molecular network are easily spotted out in the review of literature. As shown in Figure 2, HIF-1α, PTTG, VEGF, FGF-2, and MMPs (mainly MMP-2 and MMP-9) are core molecules responsible for invasiveness owing to their ability to directly or indirectly induce cell proliferation, EMT, angiogenesis, degradation, and remodeling of ECM. HIF-1α induced by hypoxia or apoplexy inside the adenoma might be the initiating factor of invasive transformation, followed with angiogenesis for overexpressed VEGF, EMT for overexpressed PTTG, degradation of ECM for overexpressed MMPs, creating a suitable microenvironment within the tumor. Next generation sequencing could be the next point of breakthrough for the investigation of pituitary adenomas, high throughput genomic data on methylation status, expression, copy number variance $$et al. on bulk samples and single cell level would push our understanding of the invasive pituitary adenomas to a much higher level.

Many of the studies demonstrated interactions between these molecules both *in vivo* and *in vitro*. Most of them focusing on the invasiveness of pituitary adenomas finally came down to the conclusion that a molecule or part of the network was involved. This network is far from complete, and the factors inducing the aforementioned changes are still not known.

More systemic investigations are required to fully understand the mechanisms of the invasiveness of different subtypes of pituitary adenomas. Attempts can still be made targeting one or many molecules at a time, to the invention of new drugs or testing existing chemicals on certain molecules.

## Author Contributions

XL designed the study. QY performed the research and wrote the paper. All authors listed have made a substantial, direct and intellectual contribution to the work, and approved it for publication.

### Conflict of Interest Statement

The authors declare that the research was conducted in the absence of any commercial or financial relationships that could be construed as a potential conflict of interest.
