# Corrigendum: Molecular Network Basis of Invasive Pituitary Adenoma: A Review

**DOI:** 10.3389/fendo.2019.00657

**Published:** 2019-09-24

**Authors:** Qi Yang, Xuejun Li

**Affiliations:** Department of Neurosurgery, Xiangya Hospital, Central South University, Changsha, China

**Keywords:** angiogenesis, endocrinology, invasiveness, molecular network, pituitary adenoma

In the original article, there was an error. In Gültekin's article, Matrix metalloproteinase-9 and tissue inhibitor of matrix metalloproteinase-2: Prognostic biological markers in invasive prolactinomas, he found that TIMP-2 is lowly expressed in more clinical samples from invasive pituitary adenoma, which was incorrectly cited by us to prove the opposite.

A correction has been made to the section ***Degradation and Remodeling of ECM by*** ***Matrix Metalloproteinases Family***, paragraph two:

“MMP-9 is the first matrix metalloproteinase found to have a significantly higher expression level in pituitary adenomas invaded to cavernous sinus (68). However, TIMP-1 was undetectable by immunochemistry staining in all samples (69). The correlation between MMP-9 overexpression and invasiveness of pituitary adenomas has been verified by many researchers in human pituitary adenoma specimens (70–75) as well as cell lines (76). Later studies showed that high expression levels of EMMPRIN (77, 78), MMP-2 (71, 75, 79), and MMP-14 (80, 81) and low expression levels of TIMP-2 (82, 83), TIMP-3 (82, 84), and RECK (85) were also correlated with invasiveness. There is a report that found TIMP-2 have higher expression in more patients of invasive prolactinomas then non-invasive ones (74), most of the aforementioned studies were performed on patients with prolactinoma or mixed patients of all secreting types, the contradicting results of TIMP-2 indicating that different types of pituitary adenoma might have distinct signaling pathways regarding to invasiveness. However, no statistical difference in the MMP-9 expression level between invasive and non-invasive non-functioning pituitary adenomas could be found (86).”

The authors apologize for this error and state that this does not change the scientific conclusions of the article in any way. The original article has been updated.

